# Physical Tampering Detection Using Single COTS Wi-Fi Endpoint

**DOI:** 10.3390/s21165665

**Published:** 2021-08-23

**Authors:** Poh Yuen Chan, Alexander I-Chi Lai, Pei-Yuan Wu, Ruey-Beei Wu

**Affiliations:** Department of Electrical Engineering and Graduate Institute of Communication Engineering, National Taiwan University, Taipei 10617, Taiwan; R09942151@ntu.edu.tw (P.Y.C.); alexiuslai@ntu.edu.tw (A.I.-C.L.); peiyuanwu@ntu.edu.tw (P.-Y.W.)

**Keywords:** physical tampering detection, channel state information (CSI), COTS Wi-Fi mobile device, deep neural network (DNN), single embedded antenna

## Abstract

This paper proposes a practical physical tampering detection mechanism using inexpensive commercial off-the-shelf (COTS) Wi-Fi endpoint devices with a deep neural network (DNN) on channel state information (CSI) in the Wi-Fi signals. Attributed to the DNN that identifies physical tampering events due to the multi-subcarrier characteristics in CSI, our methodology takes effect using only one COTS Wi-Fi endpoint with a single embedded antenna to detect changes in the relative orientation between the Wi-Fi infrastructure and the endpoint, in contrast to previous sophisticated, proprietary approaches. Preliminary results show that our detectors manage to achieve a 95.89% true positive rate (*TPR*) with no worse than a 4.12% false positive rate (*FPR*) in detecting physical tampering events.

## 1. Introduction

Security is highly important in Internet of things (IoT) systems especially concerning the communication architecture and the accessibility. Anyone authorised with the right credentials can access the system and make changes accordingly with respect to the level of authorisation. The pervasive deployment of Wi-Fi infrastructures in almost all indoor environments makes Wi-Fi the ubiquitous candidate as a communication protocol for IoT systems in monitoring and sensing localisation, human behaviour, surveillance, people counting, sedentary behaviour analysis, and so on [1,2,3,4,5]. The current state of the art focuses on harnessing the noninvasive, convenient, and cost-effective nature of Wi-Fi to achieve over-the-air monitoring continuously in indoor environments using radiofrequency signals and electromagnetic concepts. In the midst of pandemic, Wi-Fi has been among the communication techniques explored for digital contact tracing, as it has the largest signal coverage in most indoor environments and a nonintrusive proximity calculation can be done to detect close contact [6,7]. In fact, the method of [8] has already been deployed on university campuses for epidemic prevention.

Judging by the fact that Wi-Fi-based sensing and localisation applications are ever-expanding and becoming more appealing, security and protection against any threats to Wi-Fi infrastructures and communication should be properly addressed for optimal performance. Issues for software-based security of Wi-Fi such as eavesdropping, denial-of-service attacks, and traffic redirection have been intensively tackled and analysed across Open Systems Interconnection (OSI) layers including encryption, virtual private network (VPN), and Wi-Fi Protected Access (WPA) [9,10,11]. Accordingly, detection and protection against the physical tampering of Wi-Fi transmitters and/or receivers should be equivalently important.

Imagine that an unauthorised user in a restricted area, with malicious intent, tries to sabotage the Wi-Fi access point (AP) by moving or changing the equipment altogether. Applications such as localisation with a fingerprinting technique or a live view of Wi-Fi-based surveillance cameras may lose their functionality due to such changes in location and orientation of the Wi-Fi transmitters. Hence, it is critical to have an additional layer of physical security to detect such attacks by utilising the characteristics of modern Wi-Fi electromagnetic waves.

The orthogonal frequency division multiplexing (OFDM) modulation scheme used in modern Wi-Fi and 4G communication networks divides the spectrum band into several small and partially overlapped subcarriers to improve data transmission robustness and bandwidth efficiency. Channel state information (CSI) represents the properties of each subcarrier, specifically, the amplitude and phase of each transmitting and receiving antenna [12]. In single-transmitter and single-receiver scenarios, the receiver will be able to receive one received signal strength (RSS) value from the transmitter for each packet. In the meantime, multiple CSI data captured in the same situation can give abundant information regarding the physical environment, which is sensitive to the physical environmental changes [13,14,15,16]. Unfortunately, prior detection results were primarily obtained using sophisticated, usually carefully calibrated multiantenna arrays and software-defined radios (SDRs) with nonstandard parameters such as high bandwidth, substantially handicapping their applicability and acceptance.

This paper introduces a physical tampering detection approach using deep neural networks (DNNs) on the CSI readings from inexpensive COTS Wi-Fi devices with a single printed circuitry board (PCB) antenna, in contrast to previous proprietary approaches [17,18]. Preliminary results in detecting real-world Wi-Fi physical tampering events in this study exhibited 95.89% and 99.75% true positive rate (*TPRs*), with as low as 4.12% and 0.28% false positive rates (*FPRs*), respectively, on two brands of Wi-Fi access points (APs) based on system-on-chip (SoC) silicon, without leveraging advanced Wi-Fi features such as multiple-input multiple-output (MIMO). Moreover, even after periods of deterioration of the measured signal models, the *TPR* and *FPR* in our approach were still retained at the levels of 84.53% and 2.61%, respectively.

The remainder of the paper is organised as follows: Section 2 presents the related works of CSI; Section 3 describes our methodology; Section 4 shows the experimental setup; Section 5 exhibits the obtained results and discussion; lastly, Section 6 summarises the takeaways from our experiment.

## 2. Related Works

CSI is a fine-grained value from the physical (PHY) layer that details the amplitude and phase of each subcarrier in the radiofrequency domain. Due to its stability and diversity, the amplitude and phase of CSI were leveraged for indoor sensing and localisation applications in [19,20,21] and were showcased as valuable with a mean accuracy around 85% even in busy environments. The authors [22] found that CSI magnitude waveforms remain relatively stable at different positions, although multiple clusters can be observed as a result of fading. The authors applied a density-based clustering algorithm to select the optimal fingerprint for localisation. Some other CSI methods were presented in [2,23], which exploited the CSI amplitude and/or phase for localisation, gesture recognition, and monitoring.

MIMO-enabled Wi-Fi devices can estimate AoA using the phase difference between individual elements of the antenna array. The authors of [21] utilised the Wi-Fi MIMO antenna systems to provide a higher-dimensional location signature, as well as to showcase the spatial diversity of CSI, resulting in an accuracy of 0.95 m. With two MIMO-enabled APs in a complex small laboratory environment, the AoA estimation accuracy achieved 5.82° with a localisation accuracy of 0.66 m [24]. Furthermore, a COTS Wi-Fi device with MIMO antennas could provide an error of at most 2.5° for AoA and AoD when estimating the orientation of an MIMO-enabled Wi-Fi DUT using joint estimation of AoA and AoD and phase correction [25]. Nevertheless, an increase in the number of paths and in distance will also increase the complexity when estimating channel properties [26]. In addition to MIMO, studies are being developed for a Wi-Fi device with a single antenna receiver for localisation using joint maximum-likelihood estimation, which can achieve centimetre-level accuracy [27], and for a peer-to-peer CSI method, which can achieve decimetre-level accuracy [28].

The orientation of the Wi-Fi device heavily influences the performance of the CSI-based sensing system, as indicated in [29,30]. Most studies ruled out this variable and instead focused on the generality of the algorithm itself. Consequently, in the real-time result, e.g., in gesture recognition, the estimation may have been off and incurred a high prediction error, which is not practical for implementation without calibration [31,32].

On the other hand, the sensitivity of CSI to location and orientation may be viable in designing a system that detects changes in the physical location of the Wi-Fi Aps, which is critical in restricted areas. A prior study was able to achieve a *TPR* of 53% in a busy office environment using Intel 5300 NICs with three antennas [17], while [18] improved the detection to a *TPR* of 99.6% using USRP X310 with directional antenna. Regarding other vendors’ Wi-Fi chips, the Nexmon CSI Tool [33,34] supports CSI extraction from Broadcom-based Wi-Fi devices such as Raspberry Pi, while the Linux 802.11n CSI Tool [35] and Atheros CSI Tool [36,37] are also available.

## 3. Methodology

In the real world, the position and orientation of the Wi-Fi AP or transmitter should be fixed after installation for optimal performance and connectivity. The CSI detector should be able to capture CSI data, as well as compare and match the information with the trained database to verify the position and orientation of the AP. In real cases, the authorised professional holds the detector at the exact predetermined location and orientation to have the same CSI waveforms as during the fingerprinting phase. If, for whatever reason, the detector picks up quite different CSI readings, then it is very likely the Wi-Fi AP was physically tampered with. Hence, this paper aimed to study the feasibility of using a COTS Wi-Fi device as the detector to capture the CSI waveforms for a deep neural network (DNN) analysis instead of sophisticated, proprietary hardware.

**Figure 1:**
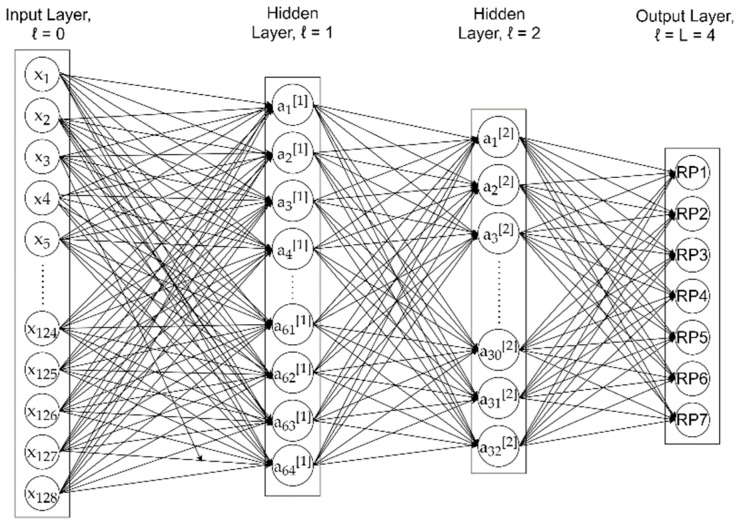
Architecture of our DNN model with two hidden layers.

Our DNN model was a fully connected neural network with two hidden layers, as depicted in Figure 1. The input layer was a 128-dimensional vector, with each element corresponding to one of the 128 subcarrier amplitudes over 40 MHz bandwidth used in the experiment [38]. Therefore, the input vector ***x*** for one training sample was

(1) $$x=\left[\begin{array}{c} x_{1}\\ x_{2}\\ \vdots \\ \vdots \\ x_{127}\\ x_{n_{0}} \end{array}\right],$$

where $n_{0}$ is the number of features in input layer 0.

For the experiment, matrix X represents all input training samples with dimensions of $n_{0}$ by m*,* where m is the number of training samples.

(2) $$X=\left[\begin{array}{cccc} x_{1}^{\left(1\right)} & x_{1}^{\left(2\right)} & \cdots & x_{1}^{\left(m\right)}\\ x_{2}^{\left(1\right)} & x_{2}^{\left(2\right)} & \cdots & x_{2}^{\left(m\right)}\\ \vdots & \vdots & \cdots & \vdots \\ \vdots & \vdots & \ddots & \vdots \\ x_{127}^{\left(1\right)} & x_{127}^{\left(2\right)} & \cdots & x_{127}^{\left(m\right)}\\ x_{n_{0}}^{\left(1\right)} & x_{n_{0}}^{\left(2\right)} & \cdots & x_{n_{0}}^{\left(m\right)} \end{array}\right].$$

The first and second hidden layers had 64 and 32 neurons, respectively, following the rule of thumb as suggested by [39] that the number of neurons in hidden layers should be between the sizes of the input and output layers, and less than twice the size of the input layer. The decreasing size of hidden layers followed the design idea in [40]. The hidden layers adopted the rectified linear unit (ReLU) activation function, while the output layer adopted the softmax operation and predicted the confidence scores among the seven RPs, indicating how likely a RP has been tampered with.

We adopted similar data collection settings to [40], which also adopted a fully connected neural network toward reading CSI signals. We collected 500 samples of CSI data while manually tampering with each of the seven RPs, amounting to 3500 samples in total with ground truth. The DNN model was trained with 2240 samples through minimising the categorical cross-entropy loss between the predicted confidence scores and the one-hot encoded ground truths, where we applied the Keras [41] Adam solver with a learning rate of 0.01 and iteration through 100 epochs.

The activation column vector $a^{\left[\ell \right]}$ in a hidden layer can be depicted as

(3) $$a^{\left[\ell \right]}=\left[\begin{array}{c} a_{1}\\ a_{2}\\ \vdots \\ \vdots \\ \vdots \\ a_{n_{\ell }} \end{array}\right],$$

where $n_{\ell }$ is the number of hidden neurons in the respective hidden layers. For hidden layer ℓ=1, it was 64, and, for hidden layer ℓ=2, it was 32.

The activation matrix $A^{\left[\ell \right]}$ for all units in each hidden layer ℓ across all the samples can be represented by

(4) $$A^{\left[\ell \right]}=\left[\begin{array}{cccc} a_{1}^{\left(1\right)} & a_{1}^{\left(2\right)} & \cdots & a_{1}^{\left(m\right)}\\ a_{2}^{\left(1\right)} & a_{2}^{\left(2\right)} & \cdots & a_{2}^{\left(m\right)}\\ \vdots & \vdots & \cdots & \vdots \\ \vdots & \vdots & \ddots & \vdots \\ a_{127}^{\left(1\right)} & a_{127}^{\left(2\right)} & \cdots & a_{127}^{\left(m\right)}\\ a_{n_{\ell }}^{\left(1\right)} & a_{n_{\ell }}^{\left(2\right)} & \cdots & a_{n_{\ell }}^{\left(m\right)} \end{array}\right].$$

Each hidden neuron receives a set of x as input to compute the predicted value a. The working principle is that the neuron computes a weighted average of x on the basis of its current weight vector w in addition of adding bias b, which can be represented as

(5) $$z=w_{1}x_{1}+w_{2}x_{2}+\ldots +w_{n}x_{n}+b=w^{T}x+b.$$

The result of z is passed through nonlinear activation function g. Therefore, the predicted value a for each hidden neuron is

(6) a=g(z).

As the neuron receives previously predicted value a as its input and multiples it by the corresponding weights, the overall vectorisation representations for ***z*** and ***a*** across hidden layer ℓ are

(7) $$z_{i}^{\left[\ell \right]}=w_{i}^{T}a^{\left[\ell -1\right]}+b_{i},$$

(8) $$a^{\left[\ell \right]}=g^{\left[\ell \right]}\left(z^{\left[\ell \right]}\right),$$

where i is the index of the neuron in that layer ℓ.

A matrix representation of weight W can be obtained by transposing and stacking w. A similar operation can be done for bias B and activation function A. Therefore, the matrix representations for hidden layer ℓ in the model are

(9) $$Z^{\left[\ell \right]}=W^{\left[\ell \right]}A^{\left[\ell -1\right]}+b^{\left[\ell \right]},$$

(10) $$A^{\left[\ell \right]}=g^{\left[\ell \right]}\left(Z^{\left[\ell \right]}\right).$$

Throughout the experiment, we chose Raspberry Pi 3B+ (RPi3B+) with a Broadcom BCM 43455c0 Wi-Fi chip with a single transmitter/receiver PCB antenna [42] as the mobile CSI detector. The advantage of using RPi3B+ is that calibration of the antenna is not needed, which reduces the complexity of system implementation, while its small and handheld size makes it practical to deploy for real-world usage. RPi3B+ was configured to run Raspbian Linux release Buster 2020-02-14 (Linux 4.19.97) in CSI monitoring mode to capture CSI data from the specific AP by using the Nexmon CSI tool.

For this experiment, the APs used were Synology RT2600ac and Asus RT-AX88U. The former, based on Qualcomm Atheros QCA9984 SoC, supports 802.11ac and MU-MIMO for 2.4 GHz and 5 GHz radio bands and features 4 × 4 transmitter/receiver antennas [43]. The latter, based on Broadcom BCM43684 SoC, supports 802.11ax, MU-MIMO, and OFDMA for 2.4 GHz and 5 GHz bands and features 4 × 4 transmitter/receiver antennas [44]. In the real world, it is uncommon to find homogeneous product deployment, as there are high variety of Wi-Fi transceivers across different system vendors and SoCs. Consequently, using identical COTS Wi-Fi APs cannot truly validate the practicality of the detector for real-world deployment. Furthermore, the position of each AP varies from one to another and, therefore, the extracted CSI waveforms will differ from one AP to another even when using identical APs due to environmental interference. Rationally, it was practical to use different brands of Wi-Fi APs to cross-examine the effect of different Wi-Fi SoCs on the observed CSI data and to investigate the universality of our detection method.

**Figure 2:**
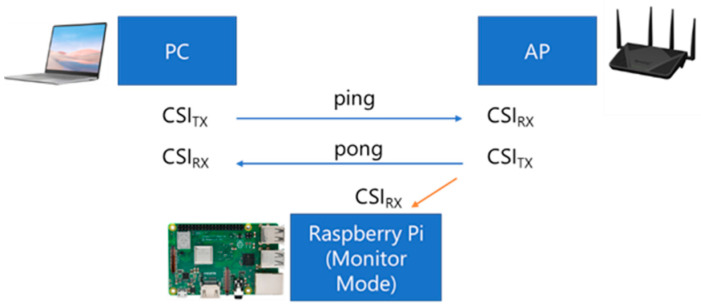
Configuration for CSI data collection in this experiment.

Meanwhile, a host PC capable of transmitting packets continuously to the AP was used to generate the packet traffic. The host PC consistently generated traffic by sending ‘ping’ requests to the target AP so that the CSI data from the AP responses could be captured and saved by RPi3B+ for further offline analysis. Figure 2 illustrates the device configuration for the experiment, adapted from [45].

## 4. Experimental Setup

The purpose of the experiment was to determine the effect of orientation of APs on the received CSI waveforms. With the assumption of software-related security concerns being out of the context of this study and that the user could not tamper with the internal hardware of the Wi-Fi device, the physical tampering events were narrowed down to moving and rotating the Wi-Fi device. It was observed that, generally, the amount of clockwise rotation of the AP is equivalent to the amount of counter-clockwise rotation of the mobile detector. For the sake of the experiment, both APs were kept constant, and only the positions and orientations of the mobile CSI detector were changed. Hence, the environment remained intact with no major changes except for the relative orientations between the AP and the mobile CSI detecting device over the course of the experiment.

**Figure 3:**
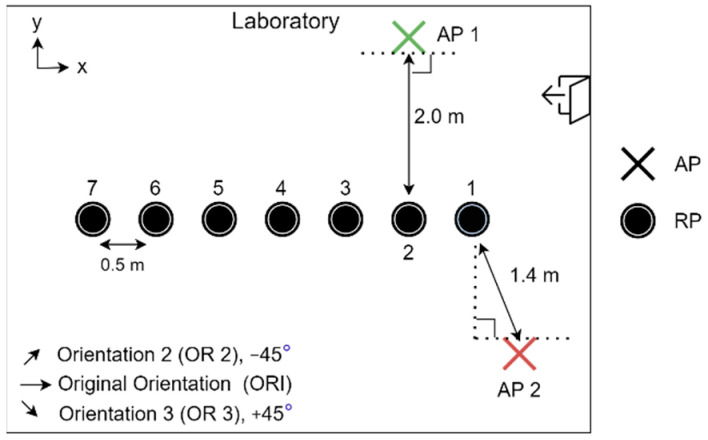
Relative locations of RPs and APs in experimental setup.

**Figure 4:**
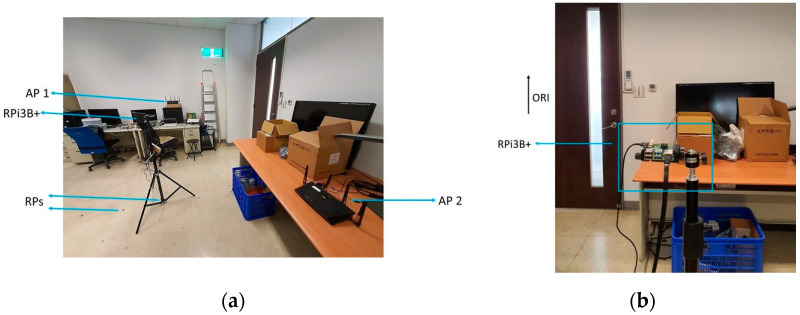
(**a**) Photo of the actual experimental environment; (**b**) photo of the setup of RPi3B+ with orientation ORI.

The experiment was conducted in the laboratory where both the Synology and the Asus APs were used one at a time. To monitor the CSI waveforms, the authorised professional held the CSI detector at predetermined RPs where the fingerprints were already collected and processed. The layout of the RPs, as well as the mounting locations of the Wi-Fi Aps, is shown in Figure 3, while a photo of the actual environment is shown in Figure 4a, and a photo of the setup of RPi3B+ with the original orientation is shown in Figure 4b. In Figure 3, the Synology AP was placed at position 1 (denoted by AP 1), and the Asus AP was placed at AP 2, both configured at channel 36 with 40 MHz bandwidth at 5 GHz.

RPi3B+, mounted at the top of a 1.2 m high tripod, captured CSI data from one AP at a time at each RP to formulate a dataset. Each dataset had seven different classes, each matching one RP. During the measurement session, RPi3B+ was placed in the same orientation, facing the same direction for all seven RPs. After successfully capturing CSI data at all RPs with the detector facing the same orientation to create the dataset, the measurement session was iterated with the detector facing another orientation to create another dataset. The same setting was repeated each day for the duration of the experiments to determine the effects of the orientation and any time-varying characteristics on the CSI waveforms for matching accuracy (*ACC*) and other evaluation metrics.

## 5. Experimental Results and Discussion

The extracted CSI data were in the form of complex numbers. MATLAB was used to extract those complex numbers from the raw *pcap* file and generate CSI amplitude and phase data in comma separated value (csv) format for each RP with a total of 3500 samples for each dataset. For this work, we did not utilise a relational database.

A Python program was used to implement our DNN model to be trained by the CSI amplitude information for further offline prediction on the *ACC* of RPs. CSI phase data were not used in the DNN, as they behaved extremely randomly and nonrepetitively across the feasible field with COTS Wi-Fi devices [5,46,47]. Supposedly, the trained model can be stored in the CSI detector in Hierarchical Data Format version 5 (HDF5) as a hierarchical structure, whereby matching of CSI data can be performed at the instant when the professional holds the detector at the predefined RP. For the preliminary work, the matching mechanism of the training dataset and testing dataset was done locally at the server instead of at the CSI detector to investigate the feasibility of the model using the same method. The stored trained model was lightweight with a size of 257 kB for 40 MHz of bandwidth, making it suitable to be deployed on an end device such as RPi3B+.

For the experiment with AP 1, the baseline Dataset 1 was used as the training dataset, whereas Dataset 2, Dataset 5, and Dataset 6 were the testing datasets with the mobile CSI detector (the RPi3B+) oriented in the same direction as in Dataset 1 with measuring times of 1 day, 3 days, and 4 days, respectively, after gathering Dataset 1. Dataset 3 and Dataset 4 were generated on the same day and in the same time period as Dataset 1, but with the orientation of the RPi3B+ detector rotating 45° to the left and right from the original one in Dataset 1, respectively.

**Figure 5:**
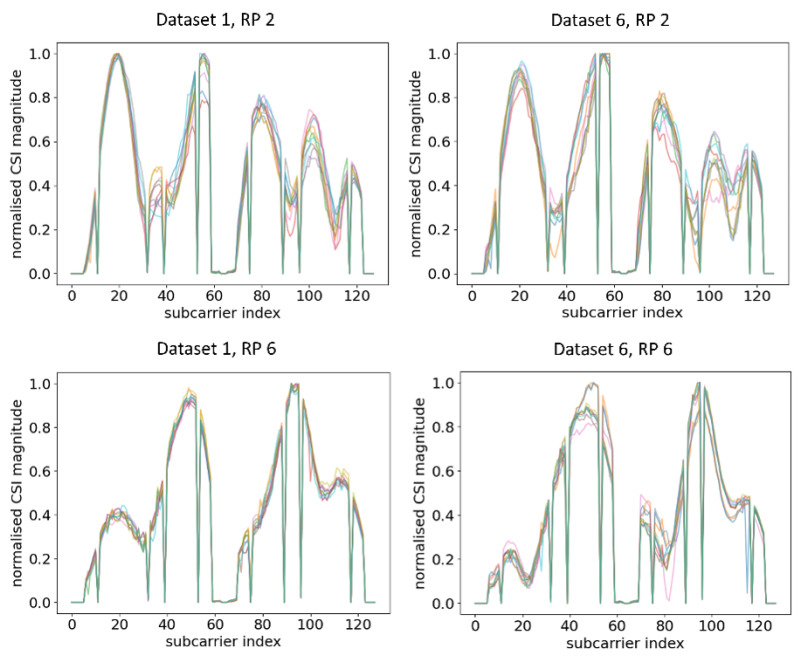
Plots of CSI amplitude during two different time periods at different RPs using AP 1, showing a similar trend in different time periods.

Ten normalised CSI amplitude waveforms for different RPs of Dataset 1 and Dataset 6 are shown in Figure 5. Even at the same RP with the same orientation of the RPi3B+, the CSI obtained can be influenced by the slightest change in indoor environment, obstacles, moving objects, and uncontrolled interference of radio waves, resulting in a different amplitude distribution of CSI subcarriers across different time periods. For example, at RP 2, there were five similar peaks for Dataset 1 and Dataset 2, while there were two similar peaks at RP 6. Evidently, all different RPs had different trends of peaks, and the trends were virtually retained even after days. The finding of our experiment resonated with [48], which evaluated the CSI amplitude at the same location with the same orientation.

**Figure 6:**
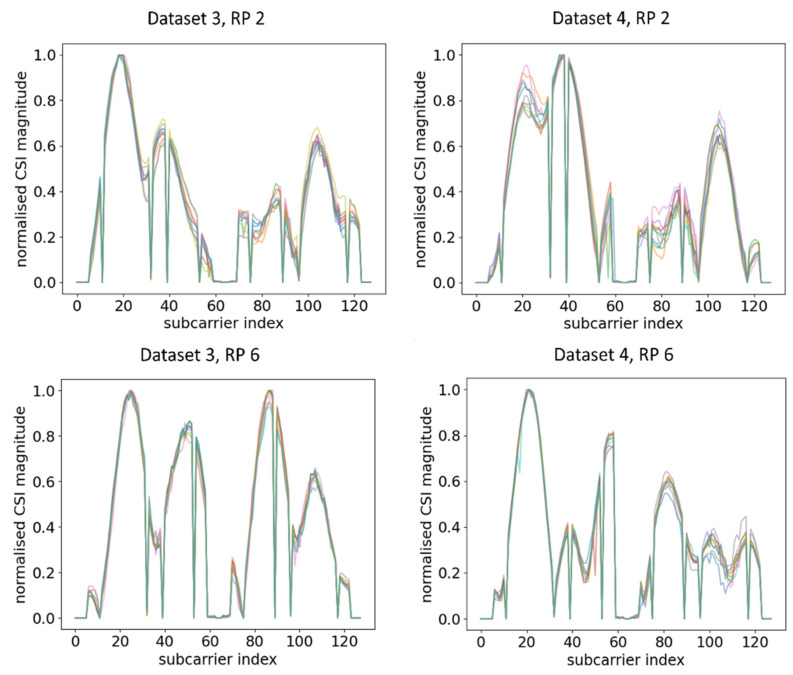
Plots of CSI amplitude in different orientations of RPi3B+ as compared with the ORI orientation in Figure 5 in the same time period at different RPs using AP 1.

Next, RPi3B+ was turned 45° left and right from the previous measurement to Orientation 2 (OR 2) and Orientation 3 (OR 3). The training data were kept the same as the previous measurement while using the datasets of the new orientation as the testing data. Note that both datasets were taken in the same time period. The CSI amplitude waveforms for different RPs across different orientations are shown in Figure 6. Figure 5 shows the amplitude with the original orientation (ORI), while Figure 6 shows the amplitude after changing the orientation.

From the comparison of Figure 5 and Figure 6, it can be observed that, even at the same RP and within the same time period under a static environment, CSI amplitudes obtained were vastly different from one another when the orientation of RPi3B+ was changed. For example, at RP6, the amplitude distributions and number of peaks were different enough across Dataset 1, Dataset 3, and Dataset 4, leading to the model recognising different patterns. A similar occurrence was identified by comparing the CSI amplitude at RP 2 in Figure 5 and Figure 6. Since the DNN with fingerprinting method works by mapping the CSI data to a geographical location, as long as the significant changes in CSI waveforms are recognisable in real time, one can infer whether physical tampering occurred.

When the system is deployed in a real scenario, our hypothetical trained professional will know exactly at which location (RP) and in which orientation they should stand to check for any physical tampering event. At exactly the same RP and in the predetermined orientation, the model will match the collected signal characteristics so that the detector will recognise it at the exact RP and in the exact orientation, thus deducing no physical tampering of the AP. Datasets 2, 5, and 6 resemble the aforementioned situation.

On the other hand, when that trained professional holds the detector in different orientations, as in the cases with Datasets 3 and 4, the signal characteristics would change significantly and the waveforms would be distinguishable from the training dataset, thus decreasing the *ACC*. As the waveforms in Dataset 1 are not transferrable to those detected, the detector can easily trigger an alert or related actions for the detected tampering event. This result shows that physical tampering detection is achievable using COTS Wi-Fi devices with a single embedded antenna.

**Figure 7:**
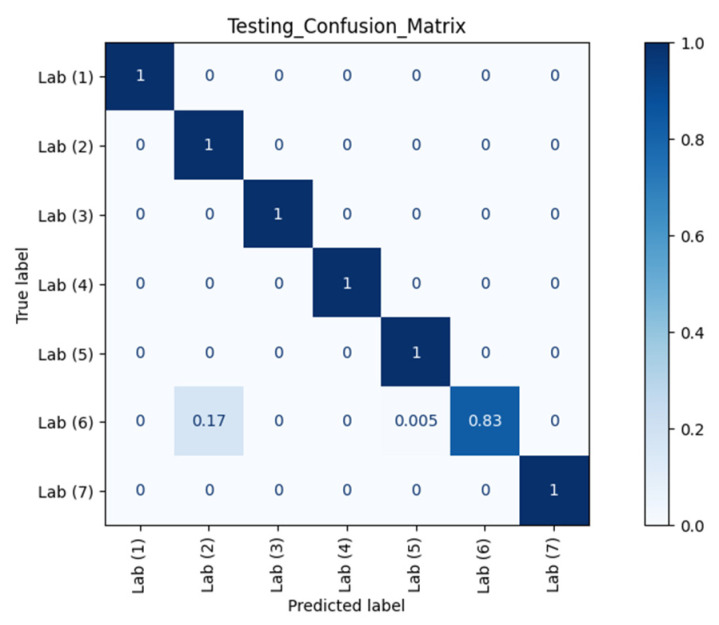
Confusion matrix of different RPs (Lab) with Dataset 2 against AP 1.

**Figure 8:**
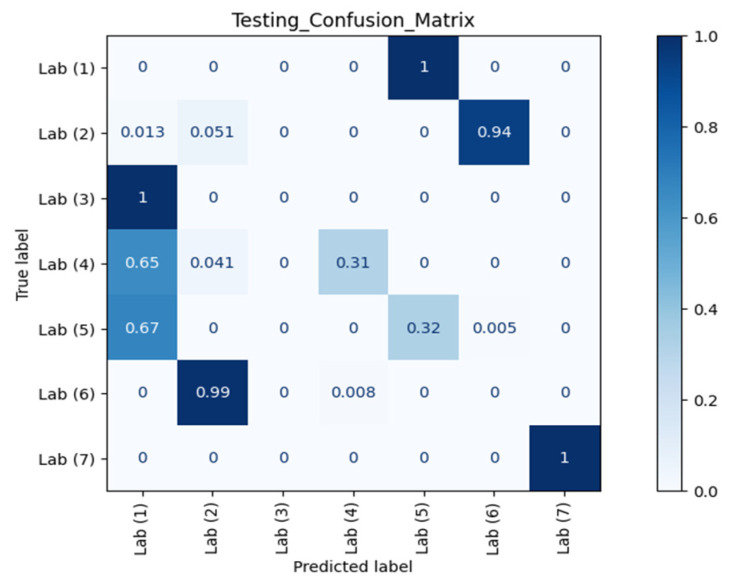
Confusion matrix of different RPs (Lab) with Dataset 3 against AP 1.

Figure 7 and Figure 8 show the confusion matrices of different RPs using Datasets 2 and 3 as the testing datasets, respectively. Clearly, our model could recognise and separate each RP when the orientation was the same as in Dataset 1, which is in line with the observation from CSI waveforms.

The performance metrics of interest were *ACC*, *TPR*, and *FPR*. *ACC* can be calculated using the following formula:

(11) $$ACC=\frac{TP+TN}{TP+TN+FP+FN}\times 100\%,$$

where TP, TN, FP, and FN stand for the probability of a true positive, true negative, false positive, and false negative, respectively. Specifically, TP is obtained when the model correctly predicts the label as the true label, TN is obtained when the model correctly predicts the label not as the true label, *FP* is obtained when the model incorrectly predicts the label as the true label, and *FN* is obtained when the model incorrectly predicts the label not as the true label. *TPR* or sensitivity can, thus, be calculated as

(12) $$TPR=\frac{TP}{TP+FN}\times 100\%,$$

whereas *FPR* or fallout can, thus, be calculated as

(13) $$FPR=\frac{FP}{FP+TN}\times 100\%.$$

The *ACC*, *TPR*, and *FPR* results at different RPs across different orientations and different time periods against AP 1 are summarised in Table 1. *TPR* or sensitivity is important for detecting any physical tampering so that as many as possible positive instances will be correctly recognised and identified and as few as possible positive cases will go unnoticed. The macro average of *TPR* from the confusion matrix of Dataset 2 in Figure 7 was 98.08% with an *FPR* as low as 0.42%. Meanwhile, from the confusion matrix of Dataset 3 in Figure 8 with different orientation, the model at RP 6 even successfully identified with 100.00% accuracy that a change in orientation occurred.

Overall, the *TPR* obtained with the testing datasets of the same orientation was at least 95.89%, while the *FPR* was no worse than 4.12%. Moreover, after 96 h (4 days), the model still retained 84.53% *TPR* and 2.61% *FPR*. Evidently, even though the *ACC* deteriorated over time, our detector model still held an adequately high *TPR* and low *FPR* as it could still recognise whether the physical Wi-Fi infrastructure was altered.

**Figure 9:**
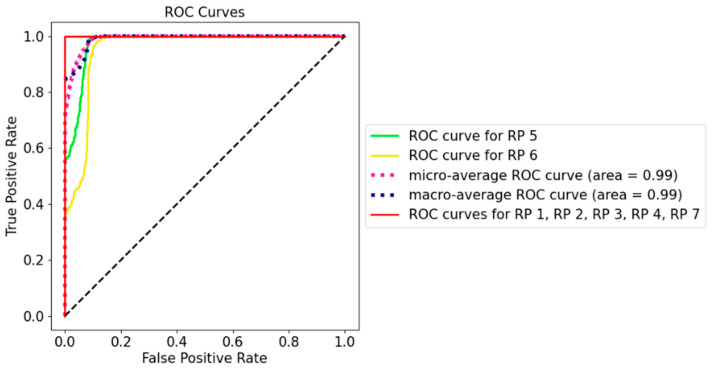
ROC curve for Dataset 6 using AP1 (the Synology AP).

The receiver operating characteristic (ROC) curve in Figure 9 measures the performance of the classification model at different threshold settings. Generally speaking, the optimal threshold was closer to the top left of the curve, which maximises *TPR* and minimises low *FPR*. According to Figure 9, the model clearly has a good measure of separability as all the classes had the curves well above the line of no discrimination, which could successfully distinguish the RPs with the same orientation. For example, even for RP 6 with an area under the curve as low as 0.95, the model could correctly label 80.00% of the cases, with a false alarm being lower than 10.00%. The curve indicated that, even after a few days, the model was still sensitive to the position and orientation of RPi3B+, and this can be translated to the real-world scenario in that, if the physical manipulation occurs at the AP, the model would still perform well in detecting physical tampering.

Next, we go one step further by investigating the influence of different Wi-Fi AP models with different kinds of SoCs. We reiterated the experiment using another AP, namely, the Broadcom-based Asus RT-AX88U (AP 2). Five more datasets, designated Datasets 7 through 11, were generated using AP 2 in a similar manner as in the case of AP 1. Dataset 7, the baseline dataset, was used as the training dataset. Datasets 8 and 11 were the testing datasets with the CSI detector (the RPi3B+) aimed in the same direction as Dataset 7 but measured after 1 and 3 days, respectively. Datasets 9 and 10 were generated on the same day and in the same time period as Dataset 7, but with the orientation of the RPi3B+ rotated 45° left and right from the original one in Dataset 7, respectively.

**Figure 10:**
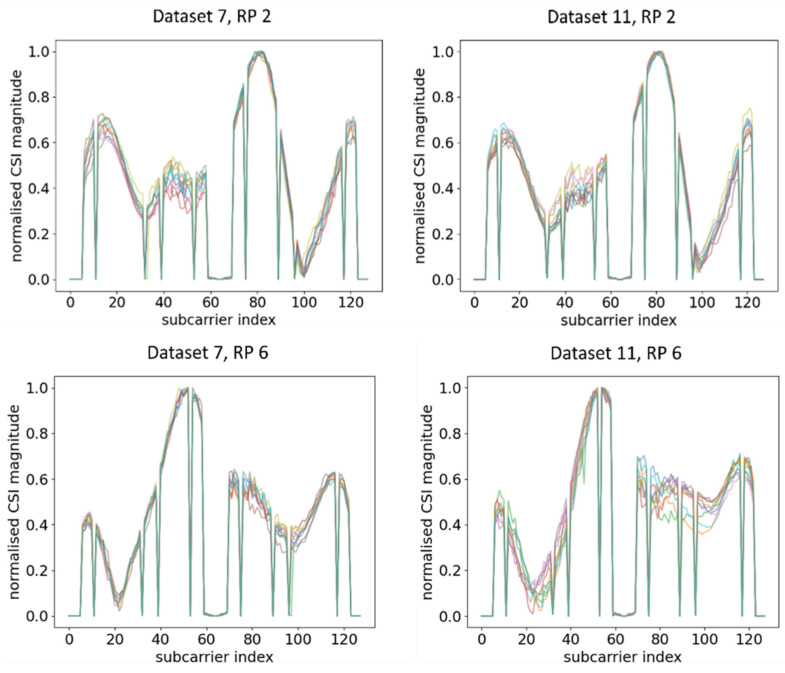
Plots of CSI amplitude in two different time periods at different RPs using AP 2.

**Figure 11:**
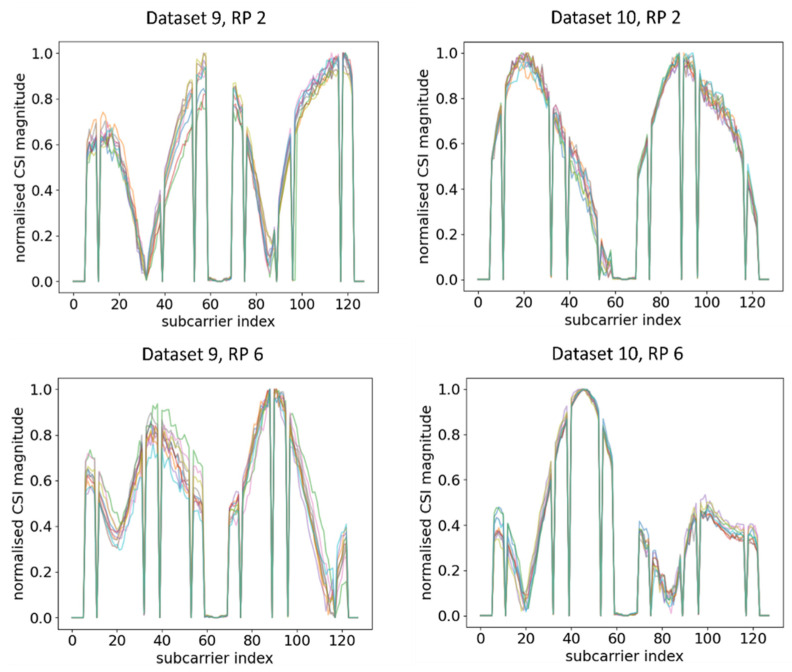
Plots of CSI amplitude in different orientations of RPi3B+ in the same time period at different RPs using AP 2.

Ten CSI amplitude waveforms of AP 2 at different RPs after normalisation are shown in Figure 10. Similar change trends of CSI amplitude across two different time periods were observed, although the deterioration was even smaller compared to AP 1. This concurred with the waveform representation method, where waveforms under two different time periods exhibited similar change trends and were transferrable. In contrast, when the orientation of RPi3B+ changed, the waveforms obtained were significantly different from the dataset with the original orientation, as shown in Figure 11 and its comparison with Figure 10.

When the relative orientation between AP 2 and the mobile CSI detector was altered from the original direction of the training dataset, the trained DNN model was still sensitive enough to detect the change, thus triggering an action for any possible physical tampering event. The evaluation metrics on different RPs across different orientations and time periods using AP 2 are summarised in Table 2. The macro average of *TPR* from Dataset 7 was 98.34% with *FPR* as low as 0.28%. Meanwhile, at RP 6 in the direction of OR 2, the detector could 100.00% correctly identify the occurrence of a physical tampering event on AP 2. The AP 2 results resonated with the observations for AP 1, which further validated the capability of simple single-antenna COTS mobile devices including RPi3B+ as a physical tampering detector, for multiple brands of Wi-Fi infrastructure devices based on various SoCs.

## 6. Conclusions

This paper recognised that the orientations of Wi-Fi transceivers have a significant effect on the captured CSI waveforms; thus, it proposed a practical real-world usage based on such a feature, i.e., physical tampering detection. The channel stationarity characteristic of CSI can be observed in which the waveforms are repetitive under the same orientation, as can be inferred from the classification accuracies by machine learning methods such as a DNN. Our preliminary approach achieved at least 95.89% *TPR* and no worse than 4.12% *FPR* in detecting physical tampering events using Wi-Fi APs from different vendors, clearly demonstrating the potential of using a COTS Wi-Fi board with a single embedded antenna, together with our proposed DNN model, as an effective physical tampering detector for Wi-Fi infrastructure without using sophisticated proprietary hardware or advanced solutions.

The time-varying trends of CSI readings were observed across various APs based on different Wi-Fi SoCs even in stationary environments. Nevertheless, our experimental results indicated that our proposed approach was relatively robust even after days of degeneration in the captured CSI signal models. In the long run, calibration and transfer learning of CSI waveforms are recommended to mitigate the declining trend of *ACC* in the event of dynamic changes and environmental noise, which hampered the tampering detection.

CSI has become part and parcel of daily Wi-Fi communication standards with the advancement of the Wi-Fi protocol; thus, future research can be performed on harvesting its potential, especially using COTS Wi-Fi devices. For example, it might be feasible to compare the results using the current implementation to real-time CSI extraction and monitoring on, e.g., modern smartphone devices, to further verify the feasibility of physical Wi-Fi infrastructure tampering detection as a smartphone IoT application. The 0.50 m resolution of RPs clearly shows the potential of this inexpensive solution in extracting CSI for successfully localisation with submeter level unit length.

## Figures and Tables

**Table 1:** Summary of evaluation metrics across different time periods and orientations on different RPs using AP 1.

Day	Dataset	*ACC*	*TPR*	*FPR*
1	Dataset 2	98.08% (average)	97.54% (average)	0.42% (average)
1	Dataset 3 (OR 2)	0.00% (RP 6)	100.00% (RP 6)	0.00% (RP 6)
1	Dataset 4 (OR 3)	4.12% (RP6)	95.89% (RP 6)	4.12% (RP 6)
3	Dataset 5	92.18% (average)	92.07% (average)	1.31% (average)
4	Dataset 6	84.43% (average)	84.53% (average)	2.61% (average)

**Table 2:** Summary of evaluation metrics across different time periods and orientations on different RPs using AP 2.

Day	Dataset	*ACC*	*TPR*	*FPR*
1	Dataset 8	98.29% (average)	98.34% (average)	0.28% (average)
1	Dataset 9 (OR 2)	0.00% (RP 6)	100.00% (RP 6)	0.00% (RP 6)
1	Dataset 10 (OR 3)	0.25% (RP6)	99.75% (RP 6)	0.25% (RP 6)
3	Dataset 11	97.89% (average)	97.98% (average)	0.35% (average)

## Data Availability

Data used to support the findings of this study are available within the article.
